# Retraction: Indigenous knowledge and quantitative ethnobotany of the Tanawal area, Lesser Western Himalayas, Pakistan

**DOI:** 10.1371/journal.pone.0331420

**Published:** 2025-09-02

**Authors:** The PLOS One Editors

The *PLOS One* Editors retract this article [1] because it was identified as one of a series of submissions for which we have concerns about competing interests and peer review integrity. Furthermore, concerns have been raised about the statistics reported in this article, the reliability of the reported results, the taxonomy used for species reported in the article, and the source and accuracy of the images presented in Fig 6. We regret that the issues were not addressed prior to the article’s publication.

The article was republished on November 29, 2023, to address an issue post-publication unrelated to the retraction of this article.

FB, ZA, NH, and RWB did not agree with the retraction. BP either did not respond directly or could not be reached.
